# Disentangling dynamic information and temporal order processing of human action perception

**DOI:** 10.1093/pnasnexus/pgaf067

**Published:** 2025-03-24

**Authors:** Vojtěch Smekal, Marta Poyo Solanas, Beatrice de Gelder

**Affiliations:** Department of Cognitive Neuroscience, Maastricht University, Oxfordlaan, Maastricht 6229EV, The Netherlands; Department of Cognitive Neuroscience, Maastricht University, Oxfordlaan, Maastricht 6229EV, The Netherlands; Department of Cognitive Neuroscience, Maastricht University, Oxfordlaan, Maastricht 6229EV, The Netherlands

## Abstract

Human action perception involves processing dynamic information in its temporal order. However, previous studies investigating action perception have not yet distinguished between the presence of dynamic information and the temporal order in which dynamic information unfolds within the context of a single action. Using 3-T functional MRI, we presented participants with brief, single-actor, whole-body actions viewed either as still images, as intact videos, or as videos consisting of short dynamic fragments with the order temporally scrambled. Regions classically associated with action perception showed higher activity for dynamic compared with still stimuli, regardless of the temporal continuity in the dynamic stimuli. However, two clusters in the right inferior parietal lobule (IPL) differentiated between the intact and temporally scrambled videos. Specifically, the right angular gyrus (AG) showed a preference for the intact videos over the temporally scrambled ones, while the right supramarginal gyrus (SMG) showed the opposite pattern. Combined with previous literature, we argue for the role of the IPL as a temporospatial buffer, with the SMG processing dynamic information on short timescales and the AG processing on longer timescales. Our results underscore the need to consider dynamic information and temporal order separately in investigations of action perception.

**Significance Statement**

Until now, the role of temporal continuity in the neural processes of action perception was poorly understood. By measuring the effects of disrupting temporal continuity in short, single-action videos, we show the central role of the right inferior parietal lobule (IPL) in temporal continuity processing. Specifically, the supramarginal gyrus shows processing of temporally scrambled stimuli, while the angular gyrus shows greater activity for temporally continuous stimuli. We thus show evidence for an anterior–posterior axis of timescale within the IPL, as well as the lateralization of temporal processing to the right hemisphere.

## Introduction

When interacting with another person, we receive a continuous stream of information, typically including their movements, facial expressions, speech, and eye gaze. This information is dynamic and occurs in a specific temporal order—to make sense of an occurring movement, the perceptual system needs to link incoming information to what is predicted based on the preceding information (1–3). If a person's raised hand moves to the left, knowing it moved to the right just before helps you recognize the action of someone waving to you. In other words, action perception involves not only the processing of dynamic movements but also how these dynamic movements are organized over time. Indeed, in a previous study (4), we have shown that temporal scrambling, or disrupting the natural order of the movements in an action, can significantly impair successful action recognition. This suggests that the integration of temporal information forms an important part of the perceptual process, in which incoming visual information about movements is transformed into an understanding of which action is being performed. While neuroscientific research has investigated various aspects of action perception, including its hierarchical organization (5), whole-body posture and movement perception (6–8), and action recognition (9), there have only been a few investigations of the importance of temporal continuity and the neural basis of its processing for action perception.

Russ et al. (10) recently focused on this question of temporal continuity by presenting macaques with stimuli scrambled to disrupt natural continuity while recording the activity of face patch neurons in the inferior temporal cortex. The authors found significantly different responses when the stimuli were presented in their natural, continuous form compared with a temporally randomized sequence, highlighting the importance of temporal context in action processing. In a human functional MRI (fMRI) study, Downing et al. (11) showed participants sequences of still images depicting simple actions either performed in their natural order or with the image sequence scrambled to disrupt the temporal continuity of the action. Regions including the right parietal cortex and left occipitotemporal cortex showed greater activity for the incoherent condition. Additionally, in a very similar study, Han et al. (12) found only regions in the right hemisphere showing greater activity for natural order sequences, with no regions displaying a preference for the disrupted order. However, these studies employed sequences of static stimuli devoid of dynamics, limiting the applicability of their findings to natural action perception, which heavily relies on dynamic information unfolding over time.

Two recent studies manipulated temporal order while also utilizing dynamic stimuli. Thomas et al. (13) used videos of complex scenarios (“actions,” e.g. preparing breakfast) consisting of different subactions termed “motor acts” (e.g. slicing the bun, spreading jam, etc.) presented either in the natural sequence or with the order of the subactions scrambled, resulting in an incoherent sequence. The authors found greater activity for the intact sequences compared with the scrambled ones in bilateral precentral and parietal clusters, while clusters in the visual cortices and temporoparietal junction showed the opposite pattern. Additionally, the higher activity in the parietal regions for the intact sequences was accompanied by a reduction in intersubject correlation (ISC) for the scrambled sequences, suggesting the regions’ inability to process the actions when temporal order is scrambled. A follow-up study using similar hand action stimuli, in combination with an ultra-high-field laminar fMRI design and connectivity analysis also showed increased activity for the intact compared with the scrambled stimuli in parietal regions (14). The authors attributed their findings in the parietal regions to feedback from premotor regions, which are involved in the representation and planning of movements. However, the stimulus manipulation consisted only in changing the order of the different subactions that together comprised the higher order action. No changes were made to the temporal order within the different subactions. Consequently, it is not possible to draw any generalizations about the impact of disrupting temporal order within a single action from their findings.

Many brain areas have been shown to be involved in action perception, including regions in temporal, parietal, and prefrontal areas—and collectively known as the action–observation network (AON; 15–17). Our previous results have shown that disrupting the temporal continuity of movements can significantly impair the recognition of associated actions (4). While past studies (18, 19) have identified parts of the AON showing greater activity for dynamic compared with static stimuli, it remains unclear, which parts of the AON are specifically responsible for the processing of temporal continuity, especially for whole-body, naturalistic, dynamic actions. The goal of this study was thus to address this aspect of action perception and to identify which regions of the brain contribute to the processing of temporal continuity of movement in the perception of single, dynamic actions. We utilized dynamic stimuli and manipulated the temporal order at the level of individual actions. One-second stimuli of single, full-body actions were presented to the participants during fMRI scanning as either still images, as videos with the temporal order intact, or as videos that had the temporal order scrambled, but still contained all the dynamic information. Unlike in previous research, this allowed us to investigate the brain basis of action dynamics and temporal continuity processing for a single action. We expected to find preferential activation throughout the cortex for dynamic stimuli compared with the static images, as well as the involvement of the parietal cortex in the processing of temporal continuity at the within-action level.

## Methods

### Participants

Twenty healthy participants (mean age: 27 years, range: 22–32 years; 10 males) were recruited for the experiment. All participants had normal or corrected-to-normal vision and no history of psychiatric or neurological disorders. They were informed about the experimental procedure, but remained unaware of the aim of the study, and provided written consent before the beginning of the experiment. Participants received gift vouchers as remuneration. The experiment was approved by the Ethics Review Committee Psychology and Neuroscience at Maastricht University and was conducted in accordance with the Declaration of Helsinki.

### Stimuli

**Fig. 1.**
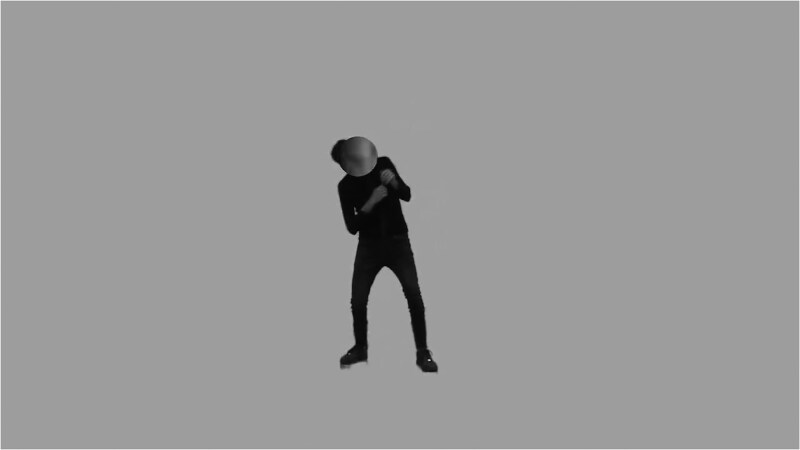
An example stimulus from the “self-protecting” action.

The stimulus set consisted of six distinct whole-body actions (self-protecting, greeting a friend, expressing frustration, brushing off, peeling a banana, and searching for an object) being performed by six different actors. The actions were selected to represent different examples from a list of putative action classes proposed by Orban et al. (20). The stimuli always contained a single actor performing the action, dressed in uniform black clothing, in front of a neutral background. Stimuli were edited to be grayscale. Actors’ faces were blurred to focus attention on the body and its movements (see Fig. 1 for an example stimulus). Detailed information about the creation and validation of the stimuli used in this study can be found in Smekal et al (4). Specifically, actions A1, A2, A3, A6, A7, and A9 from Smekal et al. (4) were used in the present experiment. The selected actions intentionally encompass a wide range, and this was also reflected in participants’ performance on a recognition forced choice task. Some actions (e.g. “peeling a banana”) were identified correctly more often than others (e.g. “expressing frustration”). Nonetheless, we showed that both the temporal scrambling procedure and showing only a static image led to a significant decrease in recognition accuracy compared with the intact video for each of them.

The stimuli were presented in three different formats: (i) as 1-s videos (frame rate = 50 Hz), (ii) as 1-s, temporally scrambled videos with the order of blocks of frames pseudorandomized, and (iii) as static images consisting of a single frame taken from each video also presented for 1 s. To create the temporally scrambled stimuli, we first defined blocks of 10 frames and then pseudorandomized the order of these five blocks. Blocks of 10 frames were chosen so as to not create too much flickering and preserve evidence of movement within the stimulus while disrupting action recognition. In a previous study (4), we showed that the scrambling procedure was effective in interfering with action recognition. The static stimuli were generated using an action–recognition algorithm (21), which selected the most representative frame from each video. The stimuli were selected from a wider stimulus set based on the results of a pilot validation study, where 81 participants categorized the action represented in 300 stimuli. For each actor and action combination used here, the videos with the highest recognition accuracy were selected (average accuracy = 90.6%).

### Experimental design

**Fig. 2.**
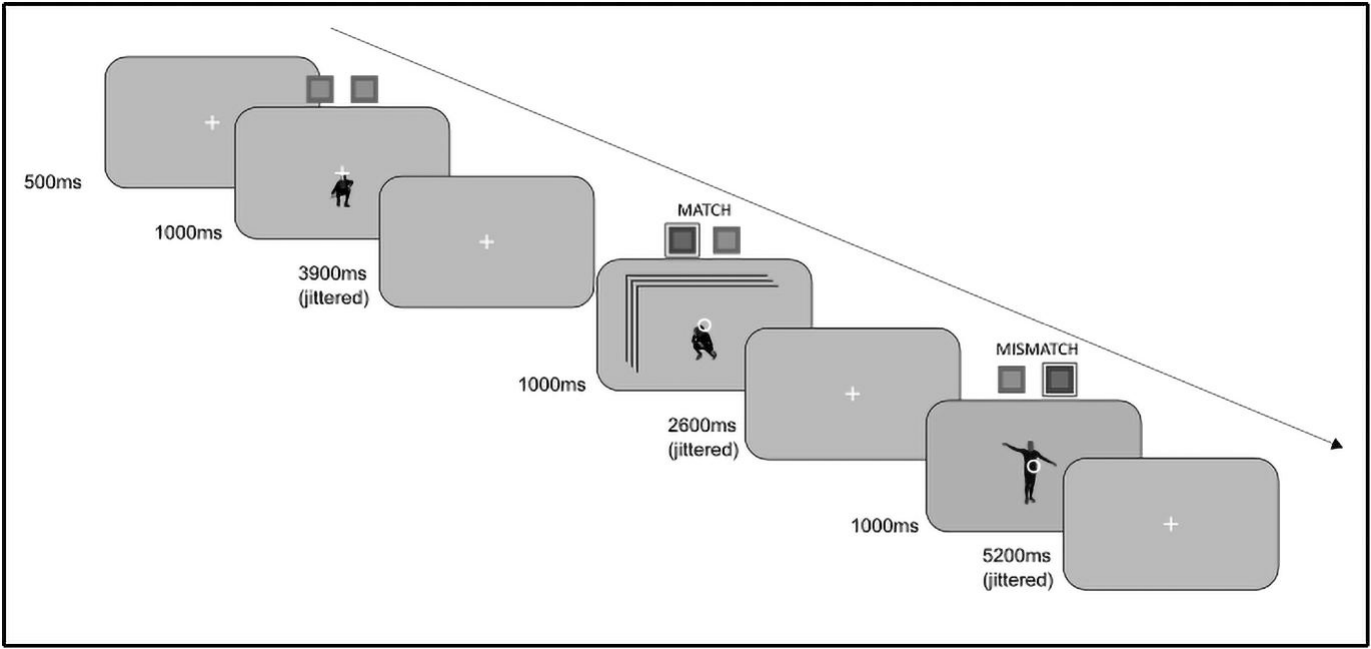
Schematic representation of the experimental procedure. Participants were instructed to respond when the fixation cross turned into a circle by pressing one of two buttons. Participants were instructed to press the left button when the action shown in the catch trial matched that of the preceding one (see the first catch trial in the diagram). Instruction to press the right button was given when the action depicted in the catch trial did not match the preceding one (second catch trial in the figure). Stimuli consisted of either still images or dynamic videos (see the first catch trial in the diagram). The figure also shows the three jittered interstimulus intervals.

The study consisted of a 2-h fMRI scanning session including the main experiment, an anatomical scan, and a functional localizer. The data from the functional localizer were not used in this study, and so they are not discussed further. The main experiment utilized an event-related design with each of the 108 stimuli (6 actors × 6 actions × 3 stimulus conditions) presented for 1 s in random order. A trial consisted of the stimulus presentation with a fixation cross overlaid, followed by a jittered interstimulus interval (2.6, 3.9, or 5.2 s) with only a fixation cross visible (Fig. 2). Each run consisted of each stimulus being presented once. The order of the stimuli was randomized in every run. Additionally, each run included an extra six catch trials, during which the fixation cross overlaid on top of the stimuli changed into a circle. When the fixation cross changed to a circle, participants performed a 1-back task, where they were instructed to press a button with the index finger of their right hand if the action matched the previously presented one and a button with their right middle finger if it was different. This task was chosen to ensure that participants attended to the stimuli without requiring them to make explicit judgments about the stimuli themselves. The total run duration was ∼10 min. The stimuli were presented with a projector onto a screen at the back of the scanner bore. The distance from the eye to the mirror was 15 cm, and mirror to the screen was 60 cm. Stimulus presentation and response recording were controlled using a custom script in MATLAB (version R2021b) with PsychToolbox (version 3.0.18; 22–24). Participants completed eight runs of the task.

### (f)MRI acquisition

The experiment was conducted using a 3-T Magnetom Prisma Fit scanner (Siemens Healtineers, Erlangen, Germany) with a 64-element head-neck coil at the Maastricht Brain Imaging Centre, Maastricht University, The Netherlands. Functional images were acquired using a $T_{2}^{*}$-weighted 2D echo-planar image sequence (number of slices per volume = 56, 2 mm isotropic resolution, repetition time [TR] = 1,300 ms, echo time [TE] = 23 ms, flip angle [FA] = 68, field of view = 1,600 × 1,600 mm^2^, matrix size = 800 × 800, multiband acceleration factor = 4). The number of volumes collected was 475 with a total scan time per run of 10.3 min. A 3D MPRAGE imaging sequence (25) was used to obtain high-resolution structural images for each participant (1 mm isotropic resolution, TR = 2,300 ms, TE = 2.98 ms, FA = 9, matrix size = 256 × 256, total scan time = 6 min).

### (f)MRI preprocessing

BrainVoyager (v22.2, BrainInnovation B.V.) along with custom scripts in MATLAB (version R2021b) using NeuroElf (v1.1; 26) was used to preprocess and analyze the fMRI data. Sinc interpolation was used to correct for time differences in slice acquisition order within one volume, and participants’ head motion was corrected with respect to the first volume of each run using trilinear/sinc estimation and interpolation. High-pass filtering was applied to exclude low-frequency noise in the data (cutoff = 3 sines/cosines). The anatomical data were corrected for B1-field inhomogeneities. For each participant, a selected functional run, closest to the anatomical run, was first aligned to the anatomical scan, creating a “dummy” volume magnetic resonance file to which all of the functional runs were then aligned. This was done to avoid the potential large misalignments that can occur during multiple direct functional-anatomical alignments. All of the data were then normalized into MNI space (27, 28). The data were spatially smoothed with a Gaussian kernel with a full-width at half-maximum of 3 mm.

The motion correction for one participant revealed motion spikes of a magnitude (>2 mm) that could not be reliably controlled for in the subsequent analyses in all their runs. Thus, the participant's data were excluded from further investigation, leaving 19 participants for the main analysis.

### fMRI analysis

The catch trials included in the experimental design were excluded from any analyses conducted on the data. Within BrainVoyager, we created design matrices for each functional run of each participant, which included each condition (6 actions × 3 stimulus conditions) as predictors, which were then convolved with a canonical two-gamma hemodynamic response function. Six *z*-transformed motion parameters were also included as confound predictors. Using these design matrices, a random-effects general linear model was fitted to the whole-brain data of all participants with individual predictors for each participant. We then used the resulting beta maps for each participant and each condition as input for the second-level random-effects analysis by running a two-factor, repeated measures ANOVA. The resulting statistical maps for the main effect of stimulus condition, the interaction effect, and the contrast of intact and scrambled stimuli were corrected for multiple comparisons using the cluster threshold estimation (Monte Carlo simulation, *n* = 5,000, alpha level = 0.05, initial *P* = 0.001), and the resulting clusters were then defined as our regions of interest (ROIs). ROIs were labeled using the BioImage Suite (29) and the Atlas of the Human Brain (30). Subsequently, the beta values for each condition were extracted from each ROI and participant, and follow-up pairwise comparisons in each area showing a significant effect were performed using SPSS to investigate the specific nature of the effect (IBM Corp., Released 2020. IBM SPSS Statistics for Windows, Version 27.0, Armonk, NY). All post hoc tests used Bonferroni correction for multiple comparisons. To further investigate the differences in the processing of temporally scrambled stimuli, the specific contrast of normal vs. scrambled videos was also conducted, which provided a greater degree of statistical sensitivity. This analysis utilized the same multiple comparison correction approach as the ANOVA analysis.

## Results

Participants performed above chance level on the one-back task (mean: 71.47%, SD: 15.99%), without showing a ceiling effect, indicating that the task was at an appropriate level of difficulty to encourage participants to attend to the stimuli.

### Stimulus condition

**Fig. 3.**
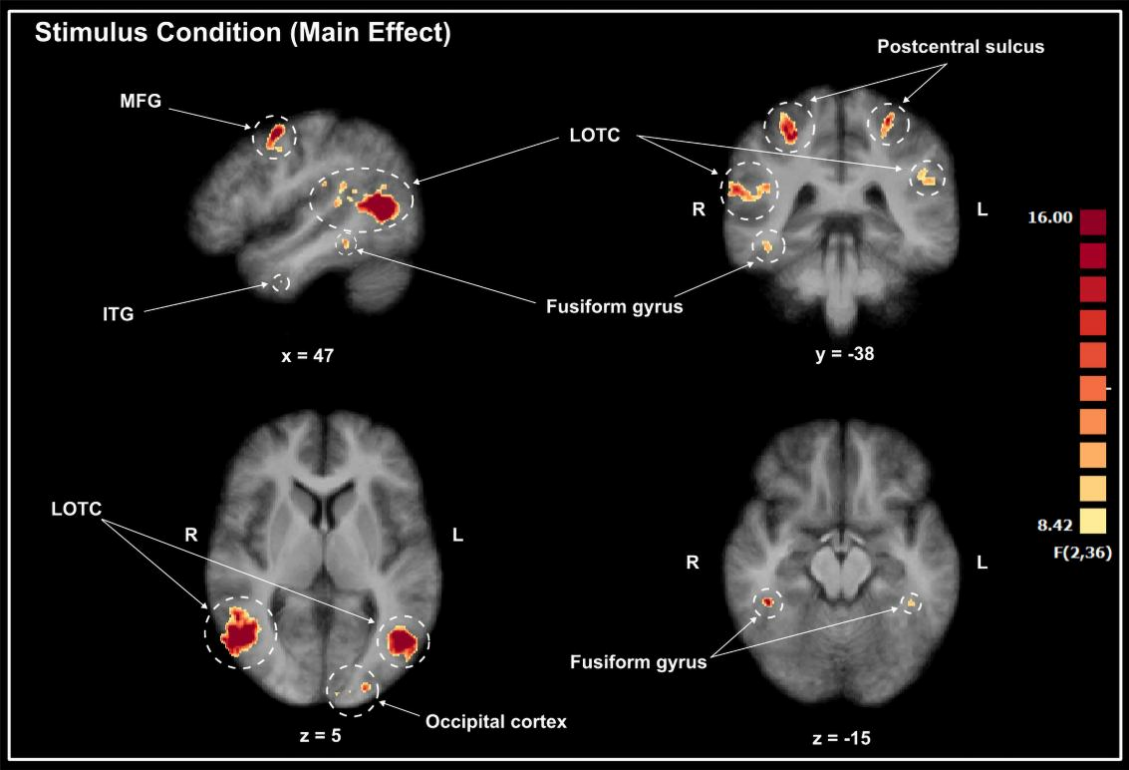
A selection of the clusters identified by the ANOVA as showing a significant main effect for stimulus presentation condition. Details about all the regions identified, cluster sizes, MNI coordinates, and statistical values are summarized in Table 1. MFG, middle frontal gyrus; ITG, inferior temporal gyrus; LOTC, lateral occipitotemporal cortex; pSTS, posterior superior temporal sulcus.

The ANOVA revealed several regions with a significant main effect of the three stimulus presentation conditions (Fig. 3 and Table 1). These regions all showed patterns of significant differences in activity between the three different stimulus conditions (still images, coherent videos, and temporally scrambled videos). Based on the results of post hoc tests, the bilateral lateral occipitotemporal cortex (LOTC), right middle frontal gyrus (MFG), right fusiform gyrus, bilateral postcentral sulcus, right precuneus, bilateral cingulate cortex, right postcentral gyrus, right cuneus, left angular gyrus (AG), left supramarginal gyrus (SMG), and a cluster in the left occipital cortex showed greater activation for dynamic (intact + scrambled videos) compared with static stimuli. Another cluster in the left occipital cortex only showed significantly greater activity for scrambled videos compared with static images. The left fusiform gyrus and right inferior temporal gyrus showed significantly greater activity for the normal videos than for the static images. Finally, a region of the left cuneus showed the highest activity for scrambled videos, followed by normal videos, and lastly static images, with significant differences between each stimulus type.

**Table 1.** ROIs showing a significant main effect of condition type, based on the ANOVA.

Brain region	Voxel size	L/R	*x*	*y*	*z*	*F*(2, 36)	Post hoc comparisons
*Video > still and scramble > still*							
LOTC/pSTS/extrastriate cortex (BA19)	10,260	R	50	−62	9	14.208	Video > still, *P* < 0.001; Scramble > still, *P* < 0.001
	6,291	L	−49	−65	11	14.521	Video > still, *P* < 0.001; Scramble > still, *P* < 0.001
Middle frontal gyrus (BA6)	872	R	46	1	53	11.412	Video > still, *P* < 0.001; Scramble > still, *P* < 0.001
Fusiform gyrus (BA37)	1,593	R	44	−41	−16	11.444	Video > still, *P* < 0.001; Scramble > still, *P* = 0.003
Postcentral sulcus (BA1)	1,905	R	34	−36	56	12.046	Video > still, *P* < 0.001; Scramble > still, *P* < 0.001
	624	L	−28	−37	61	10.204	Video > still, *P* < 0.001; Scramble > still, *P* < 0.001
Precuneus (BA7)	72	R	12	−43	59	9.456	Video > still, *P* = 0.002; Scramble > still, *P* = 0.036
Cingulate cortex (BA6)	106	R	9	−15	44	9.992	Video > still, *P* = 0.001; Scramble > still, *P* < 0.001
	112	L	−12	−22	39	10.666	Video > still, *P* < 0.001; Scramble > still, *P* < 0.001
Postcentral gyrus (BA5)	113	R	9	−45	66	9.471	Video > still, *P* = 0.002; Scramble > still, *P =* 0.020
Occipital cortices (BA18)	1,087	L	−17	−97	9	10.692	Video > still, *P* < 0.001; Scramble > still, *P* < 0.001
Cuneus (BA19)	232	R	28	−83	39	9.709	Video > still, *P* = 0.016; Scramble > still, *P <* 0.001
Supramarginal gyrus (BA40)	1,520	L	−52	−32	28	11.329	Video > still, *P* < 0.001; Scramble > still, *P* < 0.001
Angular gyrus (BA39)	94	L	−52	−59	21	9.446	Video > still, *P* = 0.004; Scramble > still, *P* = 0.012
*Video > still*							
Fusiform gyrus (BA37)	80	L	−44	−42	−17	9.305	Video > still, *P* = 0.006
Inferior temporal gyrus (BA20)	274	R	43	−4	−32	9.882	Video > still, *P* = 0.001
*Scramble > still*							
Occipital cortices (BA18)	1,340	L	−16	−91	−6	11.285	Scramble > still, *P* < 0.001
*Scramble > video > still*							
Cuneus (BA19)	348	L	−22	−85	33	9.679	Video > still, *P* < 0.001; Scramble > video, *P* = 0.011; Scramble > still, *P* < 0.001

The column labeled as “Post hoc comparisons” shows the significant post hoc tests within each ROI. All *P*-values were corrected for multiple comparisons using the Bonferroni correction. L/R indicates brain hemisphere. Coordinates are in MNI space and identify the peak voxel. *F*-values represent the average statistical value of the cluster.

An additional direct comparison of intact videos and still images is available in the Supplementary material. The focus of the current report is on the stimuli presentation conditions. Other results concerning the differences between action categories will be discussed in a future manuscript.

### Interaction (stimulus condition × action type)

**Fig. 4.**
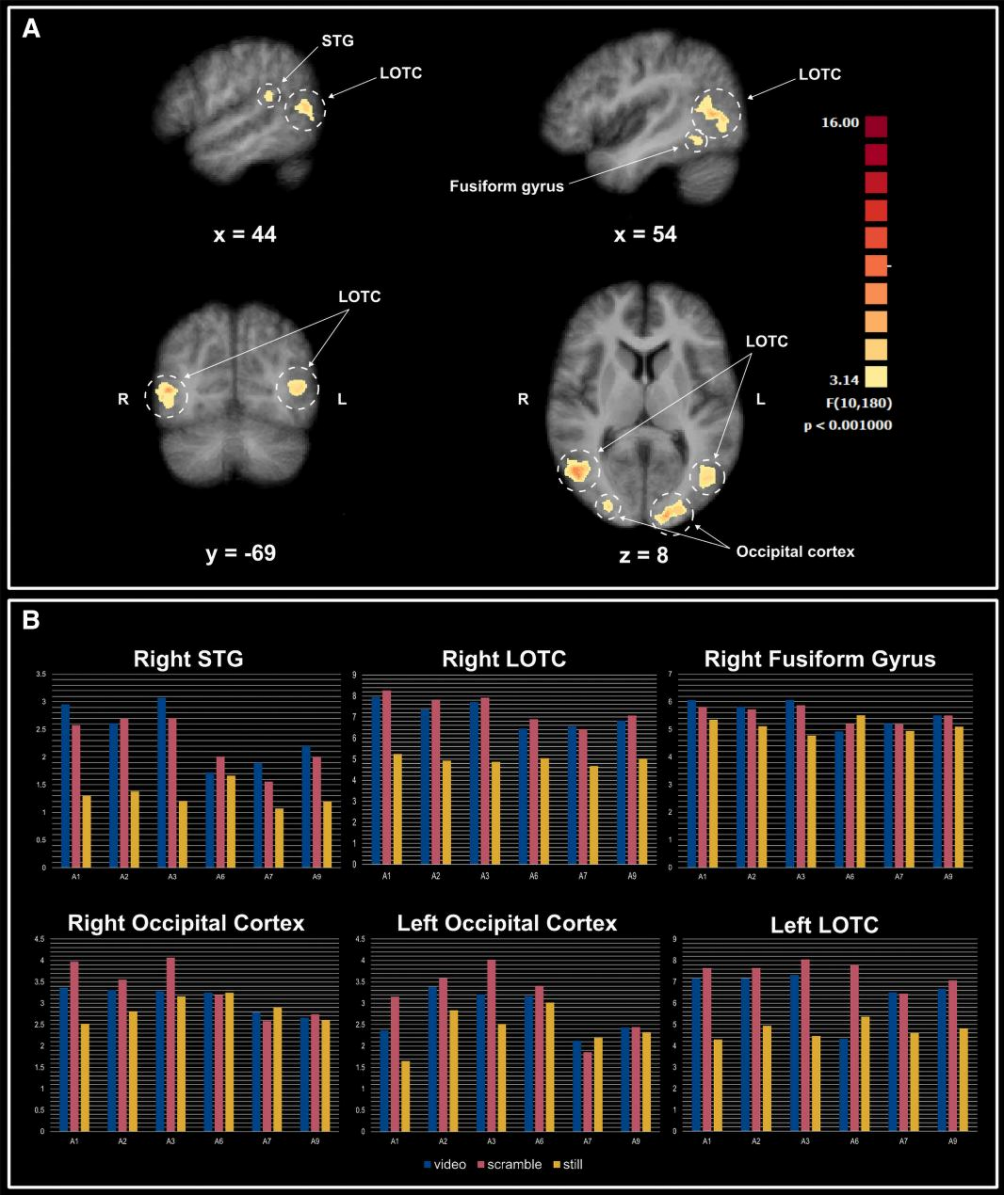
A) The clusters show a significant interaction effect between the action category and stimulus presentation condition. Details about the regions, cluster sizes, MNI coordinates, and statistical values are found in Table S1. B) The average *t* values for each action category and stimulus condition within each of the six identified clusters show a significant interaction effect. Note the differing values on the *y*-axis for each cluster. Blue represents the coherent videos, pink the incoherent videos, and yellow the still images. STG, superior temporal gyrus; LOTC, lateral occipitotemporal cortex.

Six brain regions within the occipitotemporal cortex showed a significant interaction between stimulus presentation condition and action type (see Fig. 4). These were the right superior temporal gyrus, bilateral LOTC, right fusiform gyrus, and bilateral occipital cortices. Post hoc tests did not reveal any significant effects.

### Normal vs. scrambled videos

**Fig. 5.**
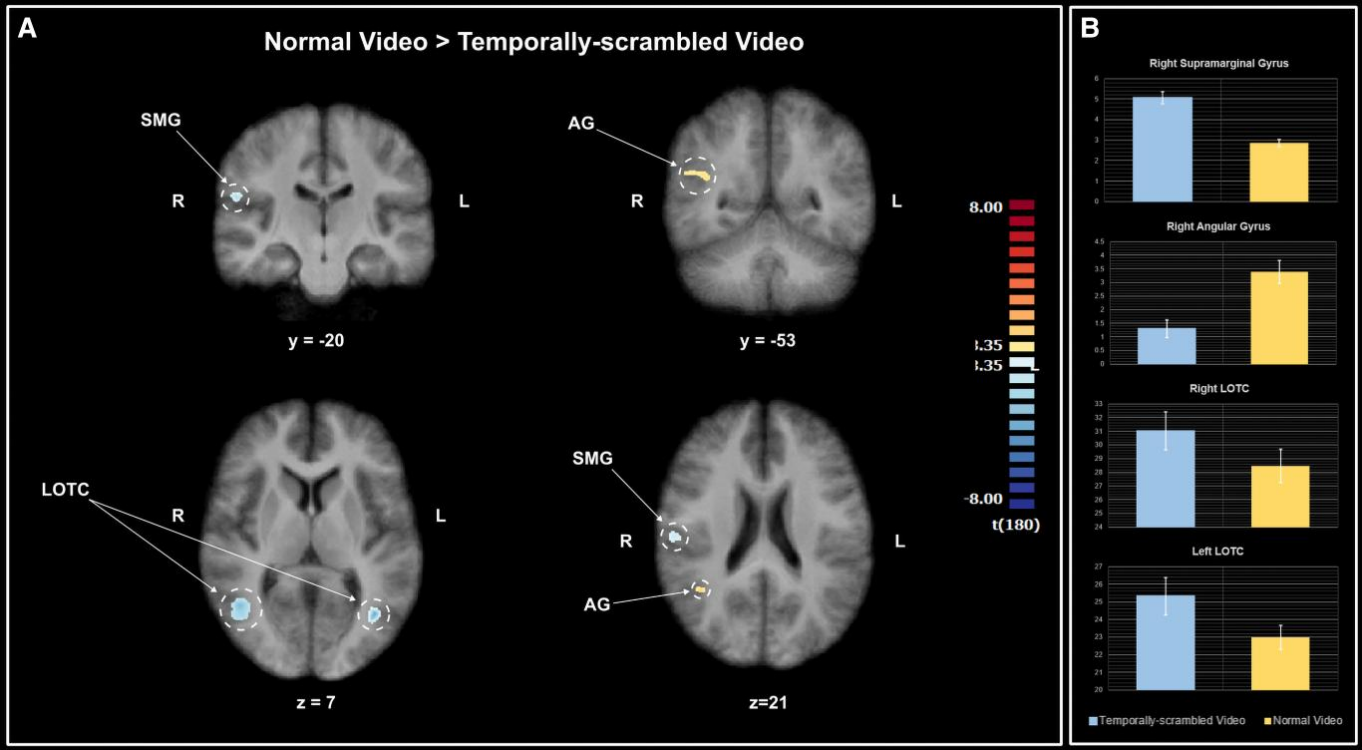
A) The clusters show a significant difference between the normal videos and the temporally scrambled videos. Both SMG and AG are regions within the IPL. Details about each cluster, including size, MNI coordinates, and statistical values, are summarized in Table 2. B) The average t-values for the normal video and temporally scrambled video conditions for each identified cluster. Note the differing values on the y-axis for each cluster. Error bars represent the SE. AG, angular gyrus; LOTC, lateral occipitotemporal cortex; SMG, supramarginal gyrus.

For greater statistical power compared with the 3 × 6 ANOVA, we also conducted a direct contrast of the coherent videos and the temporally scrambled videos. This direct comparison of normal and scrambled videos revealed a region in the right AG showed the opposite effect of greater activation for the normal videos compared with the scrambled versions. Regions in the right SMG and bilateral LOTC showed significantly greater activation for the temporally scrambled videos (Fig. 5 and Table 2). Note that these are not the same LOTC regions identified as showing a significant main effect of stimulus condition (although the two regions do overlap; see Fig. S2).

**Table 2.** Clusters identified as having a significant difference between the responses for the normal and the temporally scrambled videos.

Brain region	Voxel size	L/R	*x*	*y*	*z*	*t*(180)	Contrast
Supramarginal gyrus (BA40)	174	R	56	−19	20	−3.835	Scramble > intact, P < 0.001
Angular gyrus (BA39)	151	R	40	−53	21	3.606	Intact > scramble, P < 0.001
LOTC (BA19)	1026	R	45	−65	−25	−4.425	Scramble > intact, P < 0.001
LOTC (BA19)	538	L	−41	−65	7	−4.062	Scramble > intact, P < 0.001

L/R indicates brain hemisphere. Coordinates are in MNI space and identify the peak voxel in each cluster. All *P*-values are corrected for multiple comparisons using the Bonferroni correction. t-values represent the average statistical value of the cluster.

## Discussion

In this study, we investigated the neural substrates of dynamic action perception with a specific focus on the processing of temporal continuity, a key aspect of action understanding (4, 10, 31, 32). The study utilized 1 s stimuli depicting single-actor actions in three conditions: a static frame, a dynamic video with the movements occurring in their natural order, and a dynamic video with the order of blocks of frames randomized to create temporal discontinuity. There are two major findings. Firstly, core regions, previously identified as being involved in action perception, showed higher BOLD signal change for dynamic compared with static stimuli. These included the lateral occipitotemporal cortices and primary somatosensory cortices in both hemispheres, the right premotor cortex, and left parietal and occipital cortices. No region showed significantly higher activity for static stimuli compared with the dynamic ones. Secondly, comparing the neural activity in response to the intact and the temporally scrambled videos, we found a cluster in the right AG showing higher activity for the intact videos compared with the scrambled ones. In contrast, clusters in the right SMG and bilateral LOTC showed the opposite pattern of greater activation for the temporally scrambled videos compared with the intact ones. Our interaction analysis identified some potential clusters of interest; however, post hoc tests did not show any significant results. In the following, we discuss our findings on the general preference of dynamic stimuli, as well as the differentiation between the dynamic conditions in each region, in greater detail.

### Still vs. dynamic stimuli

The finding that, overall, dynamic stimuli (both intact and scrambled videos) led to higher activation than still images in areas involved in action perception is consistent with previous fMRI studies, which have specifically compared these two stimulus conditions for matching actions (18, 19, 33, 34). Grezes et al. (18) and Pichon et al. (19) compared responses with videos and static images of a person opening and closing a door in manners conveying different emotions. Their results showed greater responses to the dynamic stimuli than to the static stimuli in a large collection of areas throughout the brain, including the bilateral LOTC and postcentral sulci, right MFG (premotor cortex), and left SMG, all of which match our results. Additionally, similarly to our results, they did not find any regions showing greater responses to static compared with dynamic stimuli. We thus confirm and expand their findings for a wider repertoire of actions. Landsiedel et al. (33) focused on the predefined regions of the extrastriate body area (EBA) and posterior superior temporal sulcus (pSTS) and compared responses to dynamic and static versions of dyadic interactions, finding greater activity for the dynamic stimuli in both regions. This aligns again with our findings for the LOTC, containing both the EBA and the pSTS.

Our findings do contrast with the results of Pitcher et al. (35), who reported that category-selective areas in the lateral occipital and temporal cortices, such as the EBA and pSTS, showed greater activity for moving than static stimuli, while ventral areas, such as the fusiform body area (FBA), did not differentiate between static and dynamic stimuli. Based on these results, Pitcher et al. argued for the role of motion as an organizing principle in lateral brain areas. However, we found that regions analogous to the EBA, pSTS, and FBA as defined in the literature all differentiated between dynamic and static stimuli. Interestingly, in our results, the left FBA showed a significant difference only between the normal videos and the still images, with no significant difference between the temporally scrambled videos and either of the two other stimulus conditions.

The difference between the current results and Pitcher et al. (35) may be attributable to the stimuli used. Pitcher et al. did not use a single static stimulus, as in our study, but instead presented three successive static frames taken from the beginning, middle, and end of their dynamic stimuli. With such a condition, viewers were provided with partial but accurate temporal information for understanding the action and could presumably fill in the blanks between the snapshots. In line with this, Orgs et al. (36) investigated the perception of apparent biological motion from a series of three still images presented in quick succession and found the connectivity between the FBA and motor areas to play an important role in reconstructing human movement from static body postures. The finding in Pitcher et al. (35) of no difference between static and dynamic stimuli in FBA may be due to the fact that their static condition of three correctly ordered snapshots triggered the perception of apparent motion. Alternatively, it is also possible that in the current study, the FBA did not show activity for the static stimuli due to adaptation effects caused by the presentation time of the static images (1 s).

### The importance of temporal order in dynamic stimuli

The main focus of this study was the role of temporal order. Using a 6 × 3 ANOVA, the investigation of the main effect of stimulus condition showed a significant difference in activation for the normal and temporally scrambled videos only in the left cuneus. No other region differentiated between these two conditions, but simply showed higher activity for dynamic compared with static stimuli. A follow-up more sensitive, direct contrast of the normal and temporally scrambled videos identified four cortical regions showing a significant difference in activation for the two conditions: the right AG showing greater activity for the normal videos and the right SMG and bilateral LOTC exhibiting higher activation for the temporally scrambled videos. Both the AG and SMG are part of a region referred to as the inferior parietal lobule (IPL) and sometimes as the ventral parietal cortex. To avoid confusion, we will use the IPL label from here on. The two regions of the right IPL and the bilateral LOTC seem to distinguish between the coherent videos and the temporally scrambled ones.

There are several explanations for what could be underlying these trends in activity in the four regions. The two stimulus conditions vary in physical characteristics only in the order of the video frames, as they contain the same visual information, just in a different temporal order. Nonetheless, on a perceptual level, other differences may have played a role. The frame-scrambling procedure creates a difference in the apparent motion energy, with the frame-scrambled videos displaying greater motion. Simultaneously, and relatedly, in the frame-scrambled videos, the biological motion is disjointed and noncontinuous, which also disrupts the action being performed. Indeed, we have shown in past work (4) that actions in the frame-scrambled videos were recognized less accurately than in the coherent videos. Hence, the two stimulus conditions vary in temporal order, motion energy, the continuity of biomechanically determined movements, and interpretability. As we will now elaborate on, we propose that the activation patterns identified in the right IPL are mainly driven by the differences in the temporal order of the stimuli, while the activity in the bilateral LOTC is due to the differences in motion energy.

### Inferior parietal lobule

The identification of the IPL as involved in the processing of temporal continuity in action perception aligns well with previous findings, highlighting the involvement of the IPL in action perception (37). The right IPL has also previously been frequently implicated in the processing of temporal information. In a review paper, Battelli et al. (38) proposed a “when” pathway of input processing and highlighted the role of the right IPL as central to the computation of event order at intermediate time scales. They particularly emphasized the right lateralization of this temporal processing. For instance, Alexander et al. (39) administered transcranial magnetic stimulation over various regions of the brain while participants completed either a time or pitch judgment task. They found that only stimulation over the right posterior parietal cortex (PPC; an area overlapping with the IPL), as opposed to the left PPC or other cortical areas, led to an impairment in performance on the time judgment task. Indeed, our results support this lateralization to the right hemisphere. While the left SMG showed a greater preference for dynamic over static stimuli, only the right SMG differentiated between the intact and temporally scrambled videos with higher activity for the temporally scrambled stimuli.

The IPL is also a key part of the Parietal Unified Connectivity-based Computation (PUCC) model (40–43). This model argues that the lateral parietal cortex (LPC), which includes the IPL and the superior parietal lobule, acts as an online buffer for temporospatial information. The authors propose the processing of temporospatial information as a unifying mechanism underlying processing in the entire LPC, but argue that different subregions fulfill different roles and have different response profiles to the same input, based on the differing connectivity of the LPC subregions (40, 42). Additionally, Humphreys and Tibon (43) highlight the emergence of an anterior–posterior axis within the LPC with more anterior regions processing information on short timescales and these processing timescales becoming longer as you move posteriorly within the LPC. Aberbach-Goodman and Mukamel (44) presented participants with intact action videos, as well as versions scrambled in three different ways: at the level of motor primitives (e.g. grasping), the action subgoals (e.g. pouring the egg), and high-order goals (e.g. adding eggs to cake batter). Aligning with the PUCC model, they found anterior regions of the right IPL to process shorter timescales of actions and posterior regions to process longer timescales. This model also aligns well with our results, as the SMG, which lies more anteriorly within the LPC, showed higher activation for the temporally scrambled videos, consisting of continuous segments of roughly 200 ms, while the more posterior AG showed greater activity for the intact videos, which contained 1 s of continuous, undisrupted information.

The nature of the temporally scrambled videos also makes it likely that they trigger closer attention than the intact videos. In the temporally scrambled versions, the progression of the video is unpredictable and unexpected, possibly drawing more attention than the coherent videos. Indeed, in a recent review of the roles of the left and right IPL, Numssen et al. (45) found that the activity of the right anterior IPL, corresponding to the SMG, showed high predictive relevance for attentional reorienting. The explanations of short-timescale temporal processing and greater attentional demands for the activity of the right SMG are not mutually exclusive. We suggest that the unexpected and unpredictable nature of the temporally scrambled videos requires the online buffering during short timescales, which is processed by the right SMG. It is important to highlight here that we argue that the processing of different timescales and temporal continuity are inherently linked. By creating our scrambled stimuli, we constructed videos, which were continuous on a shorter timescale—from the 1 s duration of the intact video to five segments of 200 ms each. The process of temporal continuity disruption creates perceptual inputs, which must then be processed on shorter timescales.

The processing of temporal continuity in stimuli of human actions was also investigated by Thomas et al. (13) and Cerliani et al. (14). These studies used different stimuli conditions and found contrasting results to the present study. The authors also identified a region in the parietal cortex showing both higher activation and significant ISC in activity for the intact compared with the temporally scrambled videos. Unlike our results, they did not identify any regions within the parietal lobe showing greater ISC or greater activity for the temporally scrambled videos. But importantly, Thomas et al. (13) and Cerliani et al. (14) only scrambled the order of the component actions within a complex action sequence. Thus, all their stimuli were longer than the individual stimuli used in this study and focused on disturbance in longer timescale processing. Based on their results, Cerliani et al. (14) proposed that the activity within the parietal regions was driven by feedback signals from the premotor cortex and argued that the IPL integrated individual motor acts into meaningful sequences. Based on our results, we propose that the IPL is not integrating incoming information based on semantically meaningful sequences, but rather based on biomechanical continuity in the movements as the key to action understanding.

Results contrasting with the current ones were also reported in Downing et al. (11). A cluster in the right AG showed greater activity for the temporally scrambled videos, and the right SMG showed higher activation for the coherent stimuli, the exact opposite pattern to our results. However, there is a fundamental difference in stimuli between the two studies, as Downing et al. (11) presented a sequence of static images in coherent or incoherent order, with each image presented for 633 ms. The absence of genuine dynamic information in the stimuli used by Downing et al. makes the comparison to the current experiment difficult. A possible explanation is that the greater activity for the temporally scrambled stimuli in the right AG reflects a processing of the stimuli at a higher timescale than the 200 ms dynamic frame blocks presented in our study.

### Lateral occipitotemporal cortex

Our results also show higher activity for the temporally scrambled videos compared with the intact ones in large clusters in the bilateral LOTC. The LOTC is a large, heterogenous area consisting of regions, which have been implicated in a variety of perceptual processes (46), including body and hand perception (EBA; 7), action observation (pSTS; 47), and perception of tools (48). Most classically, the LOTC also contains the human middle temporal complex, hMT+, a motion-selective region responding to a variety of visual motion parameters (49, 50). We propose that the greater activation within the bilateral LOTC for the temporally scrambled stimuli is driven primarily by the greater movement energy within the stimuli. The scrambling procedure disjoins the body movements and causes significant “jumps” within the videos. An alternative or perhaps complementary explanation comes from the aforementioned study by Downing et al. (11). Presenting coherent and incoherent sequences of static images, the authors found that the EBA showed higher activation for the incoherent stimuli compared with the coherent stimuli. The authors proposed that EBA activity adapts to images closely aligned with the preceding input, as is the case in coherent movement sequences. This could be driven specifically by biomechanical continuity among the coherent sequences. The same explanation may apply to our stimuli and may indeed be compounded with greater activity within the hMT+ for the greater motion energy in the temporally scrambled stimuli.

### Limitations and future directions

We identified clusters showing a significant main effect of presentation type, as well as clusters displaying a significant interaction effect between action and presentation type. However, the post hoc tests conducted on the ROIs exhibiting the interaction effect yielded nonsignificant results. This may reflect insufficient statistical power rather than an absence of an interaction effect between those conditions. Given the implications of this issue for interpreting our findings, we created a map illustrating the overlap among regions with a main effect of presentation type, those with an interaction effect, and those identified in the whole-brain contrast of scrambled vs. intact videos (see Fig. S2). Although substantial overlap is observed in several regions associated with the interaction effect, the regions emphasized in the discussion section—specifically the SMG and AG—do not overlap with these interaction areas. Thus, their interpretability in relation to the role of temporal continuity remains unaffected.

An examination of the *t*-values in Fig. 4 for each stimulus condition–action combination suggests that the interaction effects may result from smaller differences between dynamic and static conditions for certain actions in some clusters compared with others. This is likely due to the inherently static nature of specific actions, where the impact of scrambling on action perception and temporal continuity is reduced. Therefore, the presence of an interaction effect does not necessarily undermine the generalizability of our findings on temporal continuity. Instead, it may reflect variability in the dynamism of the actions within our dataset, which could have contributed to the observed interaction effects.

Entirely disentangling the relationship between temporal scrambling within an action and the inherent jumps in biomechanical continuity, which this introduces, is an important future direction. As we have discussed, these “jumps” in our temporally scrambled stimuli also likely attract greater attention and make the stimuli inherently more unpredictable. A future investigation could utilize intact and temporally scrambled videos, which however both contain cuts that interrupt the biomechanical continuity. This could be achieved by simply omitting some frames in the intact videos. This would allow for greater control over the effect of attention and biomechanical continuity. Additionally, to build on this experiment and the work of Downing et al. (11), future investigations may include conditions of correct temporal order and disrupted temporal order for both dynamic and static stimuli, which would further the investigation of temporal processing independent of stimulus parameters.

## Conclusion

Temporal order of dynamic images is a core component for triggering action understanding. We identified two right-lateralized clusters in the IPL differentiating between intact and temporally scrambled videos. These stood out from other brain regions typically involved in action perception, which showed a greater preference for dynamic than static stimuli, but without distinguishing between conditions of temporal variability. We argue that these regions provide evidence for the role of the IPL in the temporospatial processing as distinguished from dynamic movement processing. Taken together, our results underscore the importance of considering dynamic information and temporal order as two separate processes in future investigations of action perception.

## Supplementary Material

pgaf067_Supplementary_Data

## Data Availability

All data can be downloaded at https://zenodo.org/records/14925831.
