# Supplementary material for: Disentangling dynamic information and temporal order processing of human action perception
Source: PNAS Nexus. 2025 Mar 24;4(3):pgaf067. doi: 10.1093/pnasnexus/pgaf067 (PMC11931616; doi:10.1093/pnasnexus/pgaf067)
Supplement: pgaf067_Supplementary_Data [file pgaf067_supplementary_data.docx]

# Supplementary Materials

| **Brain Region** | **Voxel size** | **L/R** | **x** | **y** | **z** | **F(2, 36)** | **Contrast** |
| --- | --- | --- | --- | --- | --- | --- | --- |
| Superior temporal gyrus (BA22) | 194 | R | 55 | -38 | 18 | 3.629 | Not significant |
| LOTC/pSTS (BA19) | 3188 | R | 50 | -64 | 8 | 4.341 | Not significant |
|  | 1564 | L | -46 | -70 | 7 | 4.165 | Not significant |
| Fusiform gyrus (BA37) | 270 | R | 43 | -53 | -11 | 3.506 | Not significant |
| Occipital cortex (BA18) | 3949 | L | -17 | -90 | -5 | 4.279 | Not significant |
|  | 566 | R | 26 | -90 | 8 | 3.543 | Not significant |

**Supplementary Table 1.** The brain regions identified as showing a significant interaction between the stimulus condition and action type. The cluster sizes within statistical maps were corrected with a Monte Carlo simulation (n = 5000, alpha level = 0.05, initial p = 0.001). L/R indicates brain hemisphere. Coordinates are in MNI space and represent the peak voxels for each cluster. *F*-values represent the average statistical value of the cluster.

| **Brain Region** | **Voxel size** | **L/R** | **x** | **y** | **z** | **t(180)** | **Contrast** |
| --- | --- | --- | --- | --- | --- | --- | --- |
| LOTC/pSTS (BA19) | 24070 | R | 50 | -63 | 7 | 8.195 | Intact video > still, *p* < .0001 |
|  | 16189 | L | -43 | -66 | 10 | 8.062 | Intact video > still, *p* < .0001 |
| Precentral gyrus (BA6) | 1460 | R | 45 | 0 | 53 | 4.822 | Intact video > still, *p* < .0001 |
| Fusiform gyrus (BA37) | 999 | R | 44 | -39 | -15 | 5.281 | Intact video > still, *p* < .0001 |
|  | 404 | L | -41 | -42 | -16 | 4.663 | Intact video > still, *p* < .0001 |
| Inferior temporal gyrus (BA20) | 113 | R | 46 | -15 | -24 | 4.631 | Intact video > still, *p* < .0001 |
| Postcentral gyrus (BA2) | 3292 | R | 31 | -36 | 48 | 5.336 | Intact video > still, *p* < .0001 |
|  | 977 | L | -33 | -38 | 57 | 4.444 | Intact video > still, *p* < .0001 |
| Occipital cortex (BA18) | 203 | R | 28 | -89 | 15 | 4.979 | Intact video > still, *p* < .0001 |
|  | 704 | L | -22 | -88 | 16 | 4.727 | Intact video > still, *p* < .0001 |
|  | 216 | L | -23 | -89 | 7 | 4.344 | Intact video > still, *p* < .0001 |
| Cingulate sulcus (BA4/5) | 125 | R | 17 | -44 | 53 | 4.380 | Intact video > still, *p* < .0001 |
|  | 272 | L | -11 | -20 | 41 | 4.727 | Intact video > still, *p* < .0001 |
| Intraparietal sulcus (BA7) | 232 | L | -20 | -84 | 37 | 4.372 | Intact video > still, *p* < .0001 |
| Supramarginal gyrus (BA40) | 3240 | L | -49 | -34 | 28 | 5.421 | Intact video > still, *p* < .0001 |

**Supplementary Table 2.** The brain regions identified as showing a significant difference in responses to the normal videos and the still images. The cluster sizes within statistical maps were corrected with a Monte Carlo simulation (n = 5000, alpha level = 0.05, initial p = 0.001). L/R indicates brain hemisphere. Coordinates are in MNI space and represent the peak voxels for each cluster. *t*-values represent the average statistical value of the cluster.


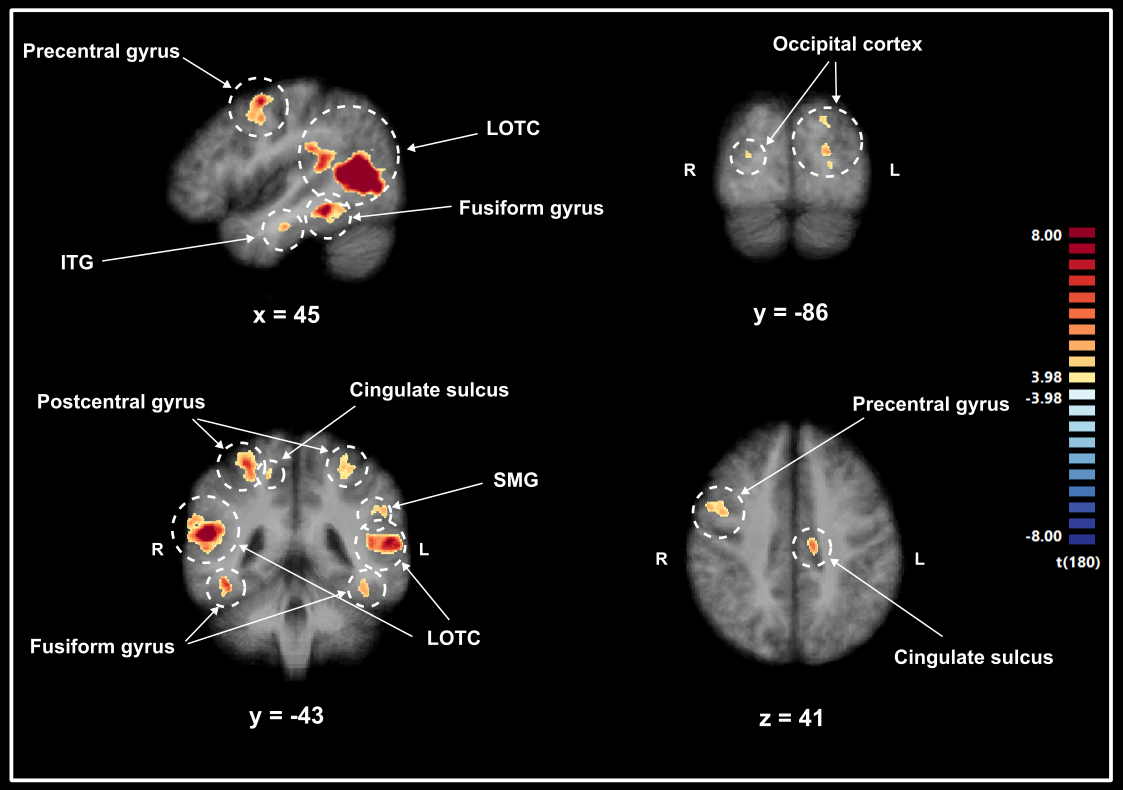


**Figure S1.** The clusters identified as showing a significant difference in activity between temporally-intact videos and still images. Details about the regions, cluster sizes, MNI coordinates, and statistical values can be found in Supplementary Table 2. Abbreviations: LOTC – lateral occipitotemporal cortex, ITG – inferior temporal gyrus, SMG – supramarginal gyrus.


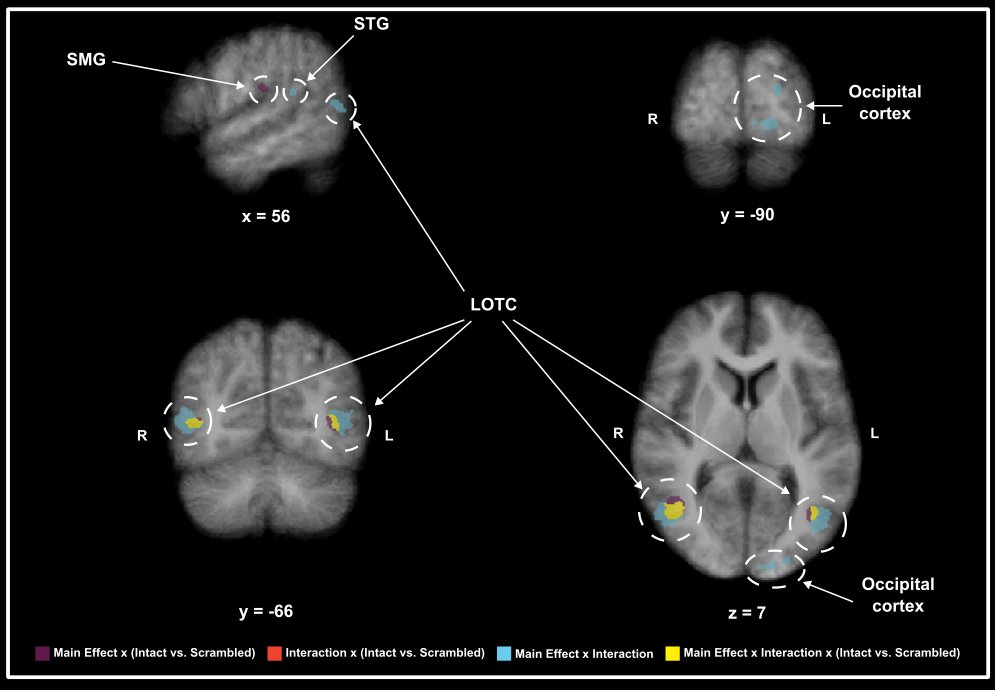


**Figure S2.** All instances of overlap between regions identified by the ANOVA main effect and interaction analyses, and the direct comparison of intact vs. scrambled videos. Overlaps occurred in the right STG and SMG, bilateral LOTC, and left occipital cortices. The colors indicate the nature of the overlap. Purple indicates an overlap between regions identified by the main effect of stimulus condition and the comparison of intact and scrambled videos. Red shows the overlap between the interaction effect of stimulus condition and action category with the direct comparison of videos. Light blue shows the overlap between the main and interaction effects. Yellow indicates an overlap between all three analyses. Abbreviations: SMG – supramarginal gyrus, STG – superior temporal gyrus, LOTC – lateral occipitotemporal cortex.
